# Detection of the stem-boring damage by pine shoot beetle (*Tomicus* spp.) to Yunan pine (*Pinus yunnanensis* Franch.) using UAV hyperspectral data

**DOI:** 10.3389/fpls.2025.1514580

**Published:** 2025-04-09

**Authors:** Meng-Ying Liu, Guang-Yun Li, Lei Shi, Ya-Ying Li, Huai Liu

**Affiliations:** ^1^ Key Laboratory of Agricultural Biosafety and Green Production of Upper Yangtze River, College of Plant Protection, Southwest University, Chongqing, China; ^2^ Institute of Highland Forest Science, Chinese Academy of Forestry, Kunming, China

**Keywords:** pine shoot beetle, stem-boring damage, hyperspectral data, Random Forest, damage level, detection

## Abstract

**Introduction:**

The stem-boring damage caused by pine shoot beetle (PSB, *Tomicus* spp.) cuts off the transmission of water and nutrients. The aggregation of beetles during the stem-boring stage results in the rapid mortality of Yunnan pines (*Pinus yunnanensis* Franch.). Timely identification and precise localization of stem-boring damage caused by PSB are crucial for removing infected wood and preventing further spread of the infestation. Unmanned airborne vehicle (UAV) hyperspectral data demonstrate great potential in assessing pest outbreaks in forested landscapes. However, there is a lack of studies investigating the application and accuracy of UAV hyperspectral data for detecting PSB stem-boring damage.

**Methods:**

In this study, we compared the differences in spectral features of healthy pines (H level), three levels of shoot-feeding damage (E, M and S levels), and the stem-boring damage (T level), and then used the Random Forest (RF) algorithm for detecting stem-boring damage by PSB.

**Results:**

The specific canopy spectral features, including red edge (such as Dr, SDr, and D711), blue edge (such as Db and SDb), and chlorophyll-related spectral indices (e.g., MCARI) were sensitive to PSB stem-boring damage. The results of RF models showed that the spectral features of first-order derivative (FD) and spectral indices (SIs) played an important role in the PSB stem-boring damage detection. Models incorporating FD bands, SIs and a combination of all variables proved more effective in detecting PSB stem-boring damage.

**Discussion:**

These findings demonstrate the potential of canopy spectral features in detecting PSB stem-boring damage, which significantly contributed to the prevention and management of PSB infestations.

## 1 Introduction

Forest ecosystems face chronic threats from human activities, climate change, pests and diseases (Boyd et al., 2013; Fei et al., 2019; Albert et al., 2023). Recently, the severity of disturbances caused by pests and diseases has increased, especially in plantation forests where monocultures are prevalent. These monocultures exhibit poor resistance to pests and diseases, rendering them highly susceptible to pest outbreaks (Naidoo et al., 2019; Lelana et al., 2022). Yunnan pine (*Pinus yunnanensis* Franch.) is an important plantation species in southwest China, playing a crucial role in ecological security, the economy, and social value (Deng et al., 2013). The pine shoot beetle (PSB, *Tomicus* spp.), a wood-boring pest, poses a major threat to Yunnan pines. Since the first outbreak in Yunnan in the last century, it has rapidly spread, damaging tens of millions of hectares of Yunnan pine forests (Kirkendall et al., 2008; Gao et al., 2012).

The damage caused by PSB is divided into two stages: the shoot-feeding and the stem-boring damage (Lieutier et al., 2003). The shoot-feeding damage caused by PSB leads to a decline in the health status of pine trees, creating conditions for stem-boring damage (Lieutier et al., 2003). The stem-boring damage caused by PSB disrupts the transportation of water and nutrients in pine trees, leading to rapid mortality of the trees (Ye et al., 2004; Lv et al., 2010). Moreover, there is also a phenomenon of aggregation of the tip-cutting borer in the stem-boring stage, where one or more types of borer are jointly damaging the same pine tree (Wu et al., 2020). In fact, multiple species of PSB infestations occur concurrently in the same forest (Lu et al., 2014). For example, *Tomicus yunnanensis* Kirkendall & Faccoli and *T. minor* Hartig collectively damage on the same Yunnan pine forest (Ye and Xue-Song, 1999; Wang et al., 2018b). In general, *T. yunnanensis* is the first to invade the host plant, followed by *T. minor*, which gradually penetrates and feeds on the stem of the tree, thereby accelerating the death of pine trees (Li et al., 2006). So, the transfer of *T. mino*r from the branches to the stems occurs approximately one to two weeks later than that of *T. yunnanensis* (Ye, 1996; Ye et al., 2004). The shoot-feeding damage mainly occurs from April to November, and the stem-boring damage occurs mainly from November to April of the following year. In particular, October to November is an important time point for PSB to transfer from branch to stem for damage, and April to May is an important time point for the newly emerged adults to transfer from stem to branch for damage (Ye et al., 2004; Chen et al., 2010; Lu et al., 2014). The shoot and stem damage of PSB complement each other, causing double damage to pine trees. Therefore, it is important to monitor the damage caused by PSB at any stage. The early monitoring of PSB damage represents an effective strategy for the prevention of stem-boring damage and the reduction of tree mortality and associated economic and ecological losses (Luo et al., 2023). The timely identification and localization of stem-boring damage is of paramount importance for the mitigation of the effects of PSB. This contributes to facilitate the clearance of infected wood, and prevent the further spread of PSB during the next stage of stem-boring damage.

Unmanned aerial vehicles (UAVs) offer flexibility and manoeuvrability, and hyperspectral remote sensing (HSR) has been demonstrated to be an effective method for detecting disturbances caused by pests and diseases (Lowe et al., 2017; Yuan et al., 2019; Zhao et al., 2022; Luo et al., 2023; Zhao et al., 2023). Consequently, the combination of UAVs and HSR technology can be employed to conduct large-area forest surveys, which provide detailed and continuous spectral information to capture subtle changes in plants. Currently, research on monitoring the damage caused by PSB mainly focuses on the shoot-feeding damage stage. At the shoot-feeding damage stage of PSB, the color and functional traits of the affected branches undergo a notable transformation, which in turn cause a substantial alteration in the host plant’s canopy spectra (Wang et al., 2018a; Wang et al., 2019; Liu et al., 2020a, Liu et al., 2021). Furthermore, progressive changes in canopy spectral characteristics were observed corresponding to increasing severity levels of PSB damage, demonstrating significant spectral response patterns across different damage stages. The early monitoring of PSB shoot-feeding damage can be effectively initiated through the observation of these changes in canopy spectra. In the previous studies by Liu et al. (2020b) and Liu et al. (2021), they identified the characteristics of spectral changes in the canopy that are sensitive to the damage caused by the tip borer, such as the red edge, the green peak, and the blue edge, etc., and realized the early monitoring of the damage caused by the tip borer by means of these characteristics.

Through these efforts, they successfully achieved the detection of shoot-feeding damage caused by PSB. However, there is a lack of exploration in monitoring the stem-boring damage stage. Firstly, in terms of timing, the occurrence of stem-boring damage by PSB with the dry season climate in Yunnan, which occurs from November to April of the following year (Liu et al., 2022). As a result of the dry season climate, the overall trend of pine forest cover declined, which made it more difficult to monitor the damage caused by PSB. In addition, the identification of stem-boring damage is still conducted through manual and visual inspection and the removal of the affected tree. However, this method is prohibitively expensive in terms of human and material resources, and it is challenging to conduct comprehensive inspections of the forest interior. It remains unclear whether stem-boring damage can result in a distinctive and substantial alteration to the canopy spectrum of the host plant. Moreover, there is currently no evidence to suggest whether the stem-boring damage of PSB can be accurately detected from UAV hyperspectral data in forested areas. In April, the simultaneous occurrence of shoot-feeding and stem-boring damage makes it challenging to discern whether the canopy spectra can be employed to differentiate between the various stages of damage caused by PSB.

Therefore, in order to promptly identify and locate the stem-boring damage caused by PSB, thus preventing the spread of damage in the next damage cycle, we collected hyperspectral data from pine forests during the stem-boring damage stage and conducted monitoring experiments on stem-boring damage caused. Through this experiment, we aim to achieve the following objectives: (1) Analyze the spectral variation characteristics of tree canopies caused by stem-boring damage from PSB. (2) Compare the differences in spectral characteristics across different damage levels of PSB. (3) Assess the performance of spectral variables based on machine learning algorithms (i.e., Random Forest, RF) in evaluating stem-boring damage caused by PSB.

## 2 Materials and methods

### 2.1 Study area and ground survey

**Figure 1:**
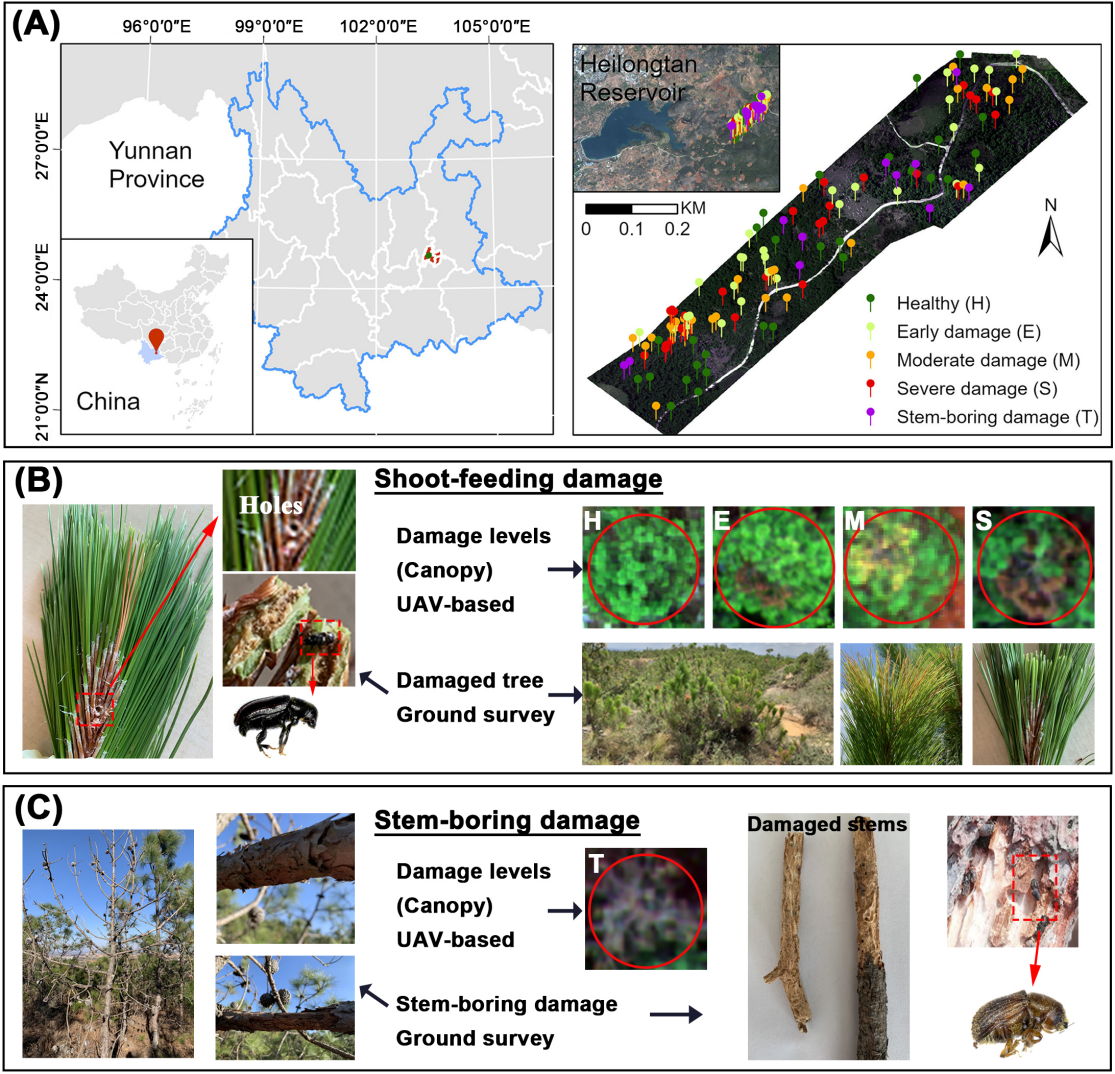
**(A)** Location of the study area and ground survey sites, **(B)** shoot-feeding damage of PSB, **(C)** stem-boring damage of PSB.

The study was conducted in a forested area near Heilongtan Reservoir, Lufu Street, Shilin County, Yunnan Province, China (24.7653°–24.7730° N, 103.3302°–103.3398° E, **Figure 1**). This area is dominated by Yunnan pine plantations, with scattered farmlands interspersed.

In April 2021, during the PSB stem-boring damage stage, we investigated PSB damage in accordance with the Standard of Forest Pest Occurrence and Disaster (Document Number: LY/T 1681–2006). UAV flights and field surveys were authorized by the Forest Pest Control and Quarantine Station of Shilin. Trees were classified into four damage levels based on canopy shoot damage rate (SDR): healthy (H, SDR ≤10%), early damaged (E, SDR 10–20%), moderately damaged (M, SDR 20–50%), and severely damaged (S, SDR >50%). Additionally, trees exhibiting stem-boring damage were classified as stem-boring damaged (T). These trees exhibited stem holes from PSB, minimal new canopy growth, and PSB galleries on branches and trunks after bark removal. A total of 122 pines (H: 28, E: 28, M: 26, S: 29, T: 11) were included in the survey. A comprehensive assessment was conducted by comparing the T level of stem-boring damage with each of the four levels (H, E, M, S) of shoot-feeding damage individually and in combination, to thoroughly evaluate the stem-boring damage of PSB.

### 2.2 Hyperspectral data acquisition

We collected pine canopy spectral data using a GaiaSky-Mini2 hyperspectral imager mounted on a DJI M600 Pro UAV. This image captures electromagnetic spectra within the 400–1000 nm range across 176 spectral bands, covering both visible and near-infrared regions. Data were collected on 25 April 2021, between 11 a.m. and 3 p.m., under clear, windless conditions. The UAV flew above the tree canopy at an altitude of 150 meters to measure canopy reflectance non-destructively, preserving the structure and arrangement of branches. The company of GISHERE (Yunnan, China) handled UAV data acquisition, image stitching, and calibration.

### 2.3 Feature variables extraction

Regions of interest (ROIs) corresponding to the canopy areas of the sample trees were manually delineated using ENVI software, and the average reflectance within these ROIs was recorded as the canopy reflectance. The spectral data were then processed using Savitzky-Golay smoothing (SG) and first-order derivative (FD) methods.

**Figure 2:**
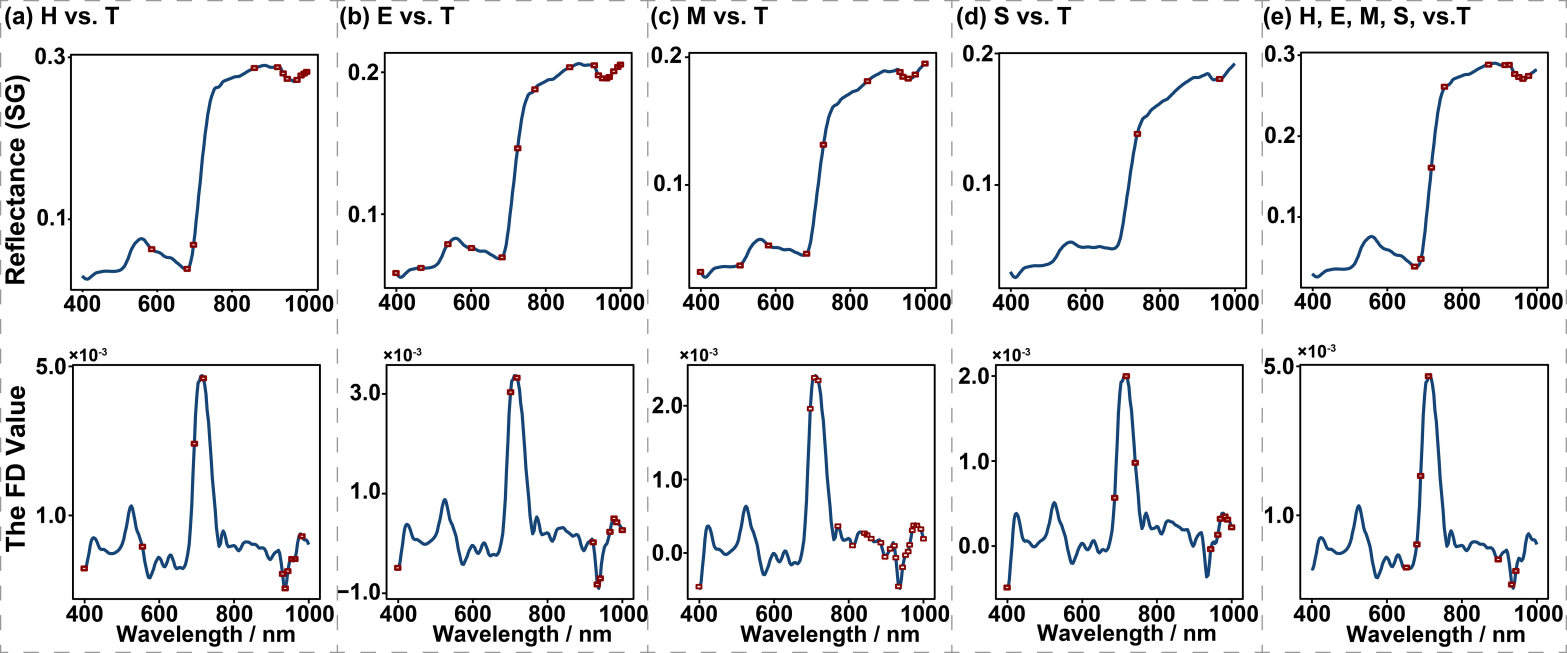
Spectral band selection of the Savitzky-Golay (SG, in the first row) and the first derivative (FD, in the second row) based on the Successive Projections Algorithm for each combination of different damage levels. The red box dots indicate the selected bands. **(a)** the SG and FD bands selected by SPA for comparative analysis between levels H and T, **(b)** the selected SG and FD bands in levels E vs. T comparisons, **(c)** the selected SG and FD bands between levels M and T, **(d)** the selected SG and FD bands in distinguishing level S from T, **(e)** the selected SG and FD bands among levels H, E, M, S, and T.

Before classifying PSB damage levels, we performed spectral feature band selection using the successive projections algorithm (SPA). The SPA is a forward iterative search method that begins with a single wavelength and sequentially adds a new variable in each iteration until the number of selected variables reaches a predefined value. The primary objective of SPA is to identify wavelengths with minimal redundancy in spectral information, thereby addressing the issue of multicollinearity (Shu et al., 2021). To compare healthy and stem-boring damaged trees (H vs. T), 11 SG bands and 10 FD bands were selected. For early damaged versus stem-boring damaged trees (E vs. T), 16 SG bands and 10 FD bands were selected. For moderately damaged versus stem-boring damaged trees (M vs. T), 11 SG bands and 24 FD bands were selected. For severely damaged versus stem-boring damaged trees (S vs. T), 2 SG bands and 10 FD bands were selected. In the combined comparison of all damage levels (H, E, M, S, vs. T), 11 SG bands and 7 FD bands were selected (**Figure 2**). The selected SG bands are named in the form “R band” and FD bands as “D band,” such as R697 and D711.

There is a strong correlation between canopy spectral information and tree health, which includes canopy structure and biochemical properties, allowing for precise estimation of tree health through spectral data (Lowe et al., 2017). Based on the spectral attributes of damaged canopies and existing literature, we identified 35 spectral indices (SIs) created from band combinations and algebraic operations (**Table 1**).

**Table 1:** Spectral indices.

Variables	Description and formula	References
Green peak Reflectance (Rg)	Maximum reflectance in the range of 520–560 nm	(Gong et al., 2002)
Red valley reflectance (Rr)	Minimum reflectance in the range of 640–680 nm	
RgP	The position of Rg	
RrP	The position of Rr	
The max FD value in blue edge (Db)	The max FD value in the range of 470–550 nm (blue edge)	
The sum of FD values of blue edge (SDb)	$\sum_{i=470}^{550}FD_{i}$ , *i* reprents the band	
The max FD value in yellow edge (Dy)	The max FD value in the range of 560–590 nm (yellow edge)	
The max FD value in near infrared (Dnir)	The max FD value in the range of 760–1000 nm (near infrared)	
The sum of FD values of yellow edge (SDy)	$\sum_{i=560}^{590}FD_{i}$ , *i* reprents the band	
The sum of FD values of near infrared (SDnir)	$\sum_{i=760}^{1000}FD_{i}$ , *i* reprents the band	
The max FD value in red edge (Dr)	The max FD value in the range of 660–740 nm (red edge)	
The sum of FD values of red edge (SDr)	$\sum_{i=660}^{740}FD_{i}$ , *i* reprents the band	
The position of red edge (DrP)	The position of the FD maximum value	
Absorption depth of red valley (D)	$1-\frac{{\text{R}}_{670}}{{\text{R}}_{560}+\frac{{\text{R}}_{760}-{\text{R}}_{560}}{760-560}\times (670-560)}$	(Wu et al., 2007)
Reflection height of green peak (H)	$1-\frac{{\text{R}}_{500}+\frac{{\text{R}}_{670}-{\text{R}}_{500}}{670-500}\times (560-500)}{{\text{R}}_{560}}$	
λmax	Difference in reflectance between the bands with the strongest positive and negative correlation	
Vogelmann red edge index (VOG)	$\frac{\left({\text{R}}_{734}-{\text{R}}_{747}\right)}{\left({\text{R}}_{715}+{\text{R}}_{726}\right)}$	(Vogelmann et al., 1993)
Red edge normalized difference vegetation index (NDVI705)	$\frac{\left({\text{R}}_{750}-{\text{R}}_{705}\right)}{\left({\text{R}}_{750}+{\text{R}}_{705}\right)}$	(Sims and Gamon, 2002)
Modified red Edge simple ratio index (mSR705)	$\frac{\left({\text{R}}_{750}-{\text{R}}_{445}\right)}{\left({\text{R}}_{705}-{\text{R}}_{445}\right)}$	
Modified NDVI705 (mNDVI705)	$\frac{\left({\text{R}}_{750}-{\text{R}}_{705}\right)}{\left({\text{R}}_{750}+{\text{R}}_{705}-2\times {\text{R}}_{445}\right)}$	
Normalized Difference Vegetation Index (NDVI)	$\frac{\left({\text{R}}_{800}-{\text{R}}_{670}\right)}{\left({\text{R}}_{800}+{\text{R}}_{670}\right)}$	(Tucker, 1979)
Simple ratio index (SR)	${\text{R}}_{800}/{\text{R}}_{680}$	(Jordan, 1969)
Green normalized differential vegetation index (GNDVI)	$\left({\text{R}}_{800}-{\text{R}}_{550})/({\text{R}}_{800}+{\text{R}}_{550}\right)$	(Gitelson et al., 1996)
Visible atmospherically resistant index (VARIgreen)	$\frac{\left({\text{R}}_{560}-{\text{R}}_{670}\right)}{\left({\text{R}}_{560}+{\text{R}}_{670}-{\text{R}}_{450}\right)}$	(Gitelson et al., 2002a)
Plant senescence reflectance index (PSRI)	$\frac{\left({\text{R}}_{680}-{\text{R}}_{500}\right)}{{\text{R}}_{750}}$	(Merzlyak et al., 1999)
Photochemical reflectance index (PRI)	$\frac{\left({\text{R}}_{570}-{\text{R}}_{531}\right)}{\left({\text{R}}_{570}+{\text{R}}_{531}\right)}$	(Gamon et al., 1992)
Enhanced vegetation index (EVI)	$\frac{5\times ({\text{R}}_{800}-{\text{R}}_{670})}{\left({\text{R}}_{800}+6\times {\text{R}}_{670}-7.5\times {\text{R}}_{475}+1\right)}$	(Liu and Huete, 1995)
Optimized soil-adjusted vegetation index (OSAVI)	$(1+0.16)\times \frac{\left({\text{R}}_{800}-{\text{R}}_{670}\right)}{\left({\text{R}}_{800}-{\text{R}}_{670}+0.16\right)}$	(Rondeaux et al., 1996)
Modified chlorophyll absorption ratio index (MCARI)	$\left[({\text{R}}_{700}-{\text{R}}_{670})-0.2({\text{R}}_{700}-{\text{R}}_{550})]\times ({\text{R}}_{700}/{\text{R}}_{670}\right)$	(Daughtry et al., 2000)
Triangular vegetation index (TVI)	$60\times ({\text{R}}_{750}-{\text{R}}_{550})-100\times ({\text{R}}_{670}-{\text{R}}_{550})$	(Broge and Leblanc, 2001)
Anthocyanin reflectance index (ARI)	$\left(1/{\text{R}}_{550})-(1/{\text{R}}_{700}\right)$	(Gitelson et al., 2001)
Carotenoid reflectance index (CRI)	$\left(1/{\text{R}}_{510})-(1/{\text{R}}_{550}\right)$	(Gitelson et al., 2002b)
Red edge chlorophyll index (CIrededge)	${\text{R}}_{\text{nir}}/{\text{R}}_{\text{rededge}}-1$	(Gitelson et al., 2005)
Water index (WI)	${\text{R}}_{900}/{\text{R}}_{970}$	(Penuelas et al., 1997)

### 2.4 Variables variance analysis

To evaluate the effectiveness of PSB damage level classification, we analyzed variance to identify spectral variables significantly different across all damage levels, particularly those highly responsive to the T level. For normally distributed variables, independent samples t-tests were used to compare two damage levels, while Duncan’s one-way ANOVA was employed to compare multiple damage levels. For variables that were not normally distributed, non-parametric tests were applied: the Mann-Whitney U test for comparing two damage levels and the Kruskal-Wallis test for comparing multiple damage levels. All samples in the dataset were included in this analysis.

### 2.5 Variables importance and modeling analysis

Machine learning methods use data to find correlations and patterns, enabling the prediction or classification of unknown data. Random Forest (RF), a supervised learning algorithm, excels in predicting future events or categories based on historical data (Khan et al., 2022). Known for its strong performance in feature classification, RF was used in this study to detect PSB damage levels. The classification algorithm based on multivariate analysis has superior performance with single variable (Nguyen and Nansen, 2020). We inputted the selected SG bands, FD bands, SIs, and their combined variables into the RF classification model, evaluating each variable’s importance using the mean decrease accuracy (MDA) index. Higher MDA values indicate greater variable importance. The model was trained on a dataset comprising all 122 samples, with accuracy assessed using the 10-fold cross-validation method. Evaluation metrics included overall accuracy (OA) and the Kappa coefficient, derived from the confusion matrix, to determine classification accuracy. The calculation formulas for OA and the Kappa coefficient are shown in Equation 1 and Equation 2. A higher OA value indicates a greater classification accuracy of the model. A Kappa coefficient below 0.4 indicates poor model consistency, 0.4–0.6 indicates moderate consistency, 0.6–0.8 indicates high consistency, and above 0.8 indicates very strong consistency.


(1) $$\text{OA}=(\sum_{\text{i}=1}^{\text{k}}{\text{TP}}_{\text{i}})/\text{N}$$

(2) $$\text{Kappa}=(\text{OA}-{\text{P}}_{\text{e}})/(1-{\text{P}}_{\text{e}})$$

Where, *TP_i_* is the number of true levels. *i* is the number of damage levels and i = 1, 2,…, *k* (*k* = 2 or 5). *N* is the total number of samples. *P_e_* is the expected agreement and Pe = ∑[(*RowTotal_i_* ×*ColumnTotal_i_*)/*N* ^2^]. *RowTotal_i_* is the sum of the *i*-th row in the confusion matrix (i.e., the total number of samples with the true level *i*). *ColumnTotal_i_* is the sum of the *i*-th column in the confusion matrix (i.e., the total number of samples predicted to be in level *i*).

**Figure 3:**
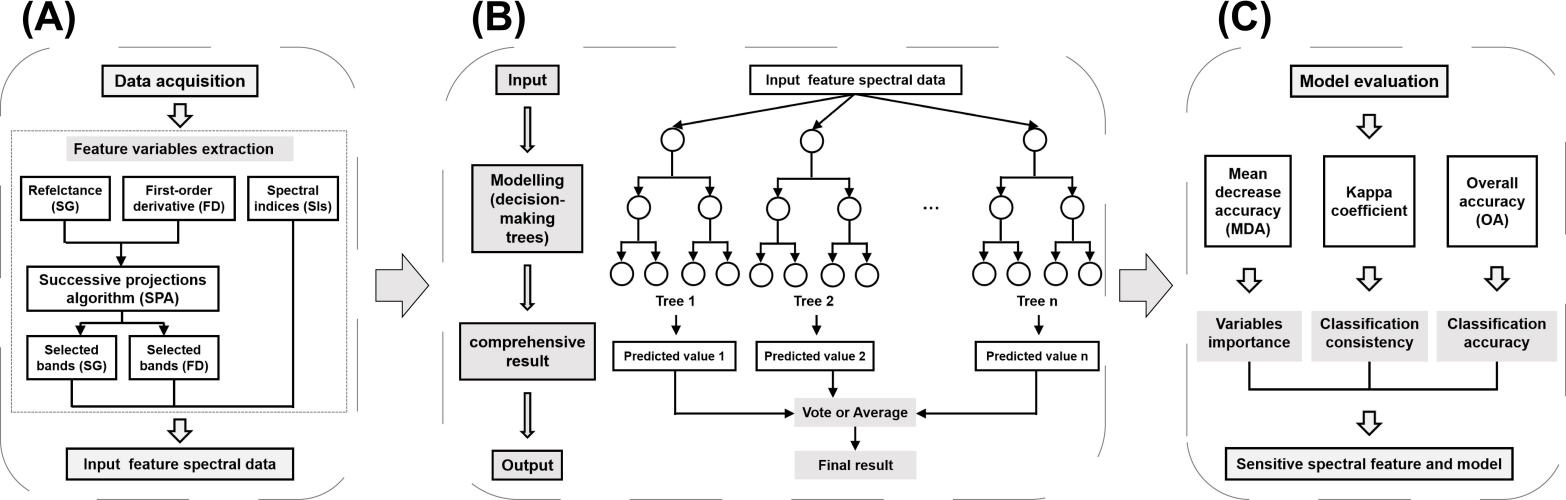
The data processing workflow. **(A)** Preparation of input data for the models; **(B)** RF model framework; **(C)** Model execution.

**Figure 3** shows the methodology and data processing workflow employed in this study consists of three main parts, including preparation of input data for the models, execution of the RF models, and evaluation of the models. During the model execution phase, we constructed four distinct categories of input datasets: (1) datasets containing only SG bands selected through SPA method, (2) datasets comprising solely FD bands selected by SPA method, (3) datasets consisting of SIs, and (4) a composite dataset integrating the aforementioned three categories. Pairwise classification was performed between damage levels H and T (H vs. T), E and T (E vs. T), M and T (M vs.T), and S and T (S vs.T), as well as classification among all five levels (H, E, M, S, vs. T). Consequently, a total of 20 RF models were executed (4 input datasets × 5 classification tasks = 20 models).

The RF algorithm is a supervised learning classification method, where each training sample is assigned a corresponding label. To ensure a comprehensive comparison, we selected Partial Least Squares Discriminant Analysis (PLS-DA), a supervised multivariate statistical analysis method, alongside Principal Component Analysis Discriminant Analysis (PCA-DA), an unsupervised learning algorithm. The effectiveness of these three classification methods was systematically evaluated and compared for detecting stem-boring damage caused by PSB. A 10-fold repetition with 10-fold cross-validation was employed. Model performance was evaluated using OA and the Kappa coefficient.

### 2.6 Discriminant analysis of PSB damage levels in different damage stages

In a previous study by Liu et al. (2020b), they analyzed the spectral characteristics of pine canopies damaged by the PSB at the shoot-feeding stage, successfully identifying the extent of PSB damage. To compare the differences between the shoot-feeding and stem-boring damage stages, we used pine canopy spectral data collected at the end of the shoot-feeding stage in November 2019 and the stem-boring stage in April 2021. The shoot-feeding stage analysis focused solely on canopy damage by PSB, categorizing trees into four damage levels (H, E, M, and S), with 20 pines in each level. These same damage levels were used for the stem-boring stage analysis. At the shoot-feeding stage, certain vegetation indices (mSR705, mNDVI705, and WI) were not calculated due to the spectrometer’s band range of 450–946 nm. The data were processed as previously described, and all samples were used for model training.

## 3 Results

### 3.1 Canopy spectral characteristics of the different PSB damage levels at the stem-boring stage

**Figure 4:**
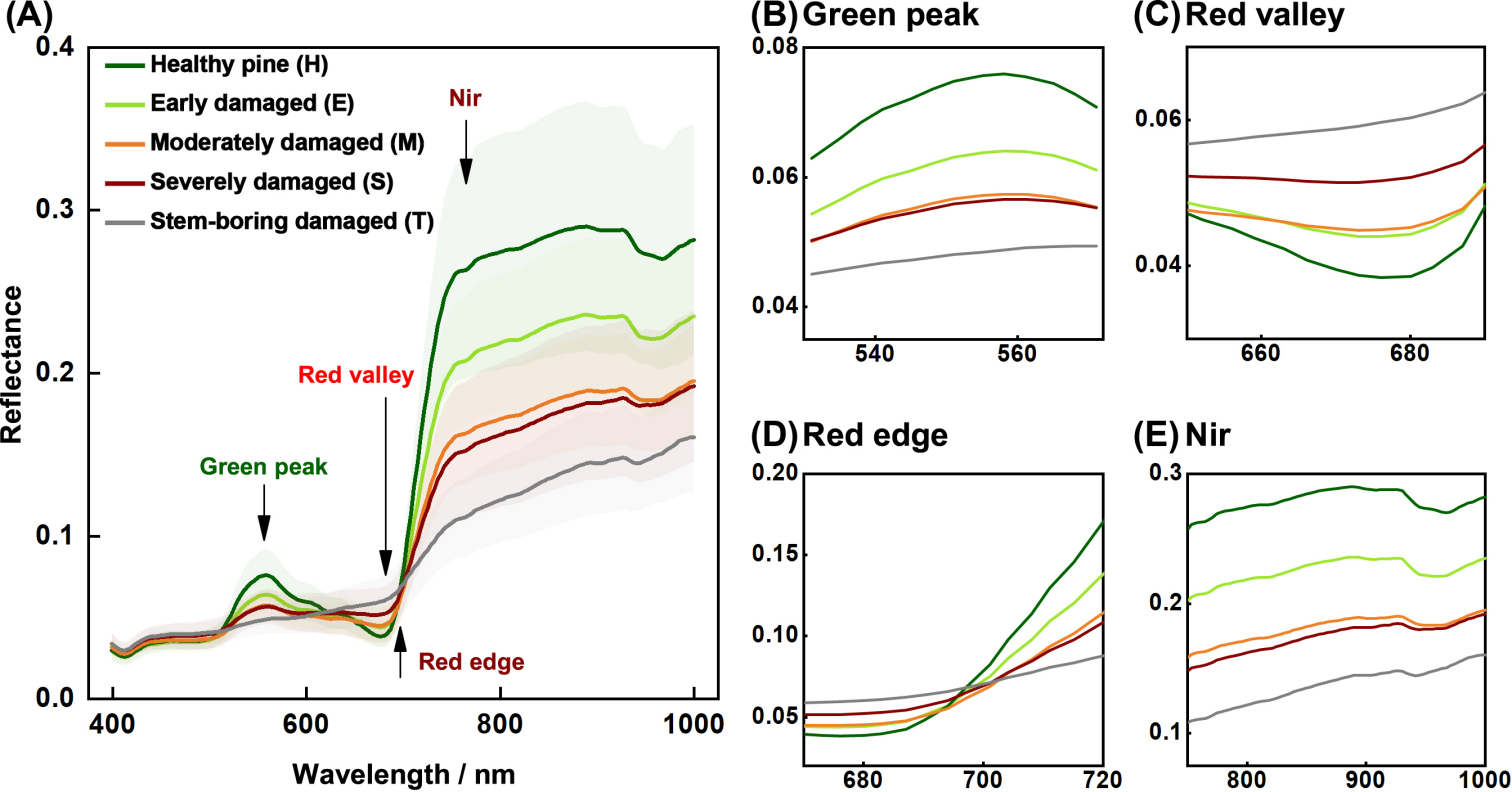
Mean canopy spectral reflectance (with 95% confidence intervals) of Yunnan pine at different damage levels. **(A)** is the whole spectral curves, and **(B)** is the rest are the green peak, **(C)** red valley, **(D)** red edge **(D)**, and **(E)** near-infrared (Nir) features, respectively.

As PSB damage severity increased, the spectral reflectance of the pine canopy decreased (**Figure 4**). Significant changes in canopy spectral reflectance due to PSB damage were observed in the green peak (520–580 nm, **Figure 4B**), red valley (650–690 nm, **Figure 4C**), red edge (680–760 nm, **Figure 4D**), and near-infrared (760–1000 nm, **Figure 4E**) regions. Damaged pines (E, M, S, and T levels) exhibited lower spectral reflectance in the green peak, red edge, and near-infrared regions compared to healthy pines (H level), with reflectance decreasing as damage severity increased. However, in the red valley, particularly within the 730–760 nm range, the reflectance of damaged canopies showed an increasing trend.

### 3.2 The variance analysis of spectral features for PSB damage levels

**Figure 5:**
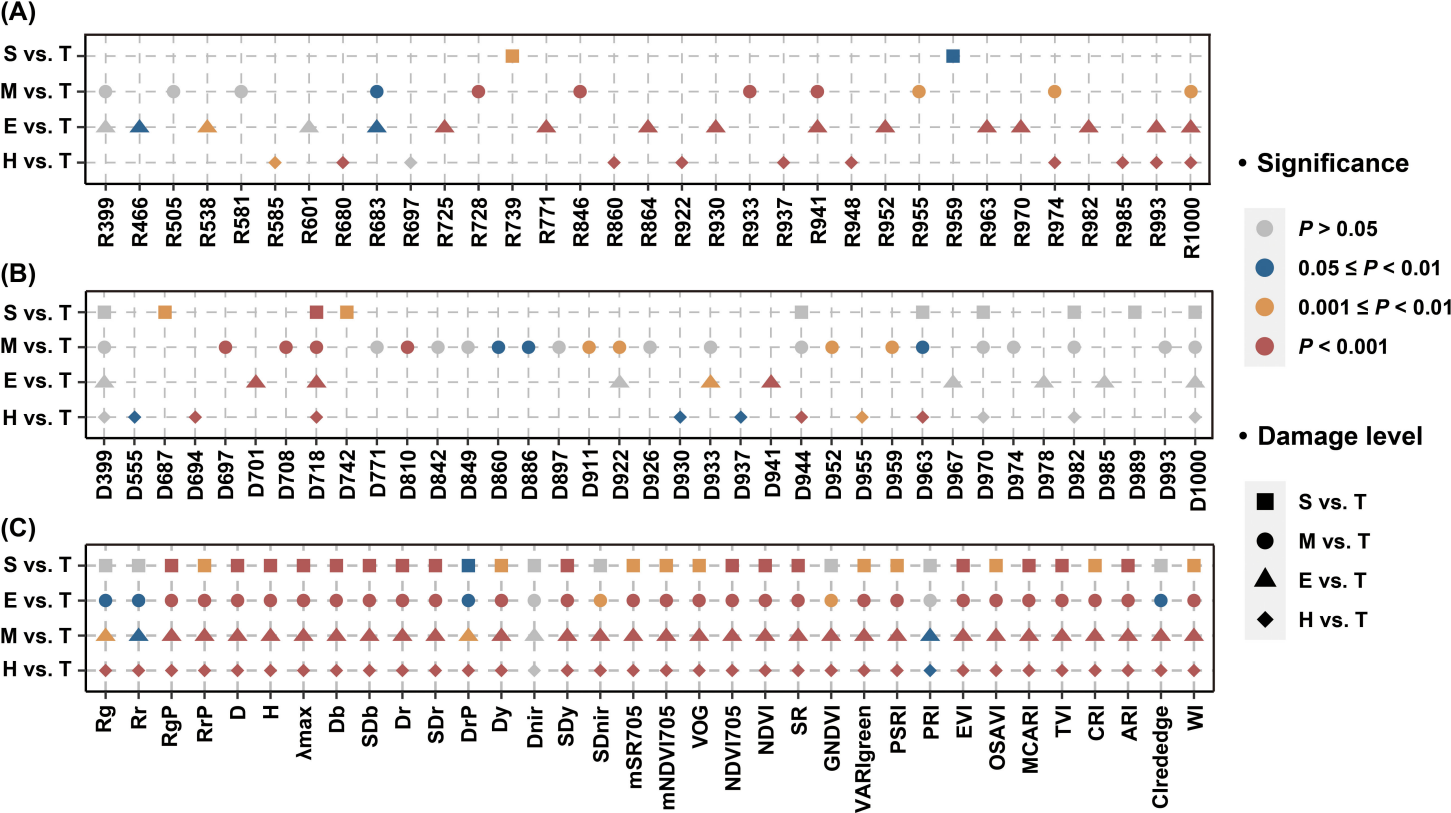
Analysis of variance for individual variable (H, E, M, and S damage levels compared to T level, respectively). The results of the variance analysis based on SG bands **(A)**, FD bands **(B)** and SIs **(C)** are shown separately.

The analysis of SG and FD bands showed that not all bands selected by the SPA algorithm were sensitive to stem-boring damage, with some showing no significant differences between damage levels (**Figures 5A, B**). Notably, the variable D718 significantly differed in all comparisons (**Figure 5B**). The potential of SIs for detecting stem-boring damage was also evaluated, with nearly half showing significant differences (**Figure 5C**). The significance of differences in some variables decreased as the PSB damage levels involved in the analysis got closer to the T level. For example, variables such as Rg, Rr, DrP, and CIrededge gradually decreased differences. The spectral indices corresponding to blue edge (e.g., Db and SDb), red edge (e.g., Dr), and yellow edge (such as SDy) positions, as well as those reflecting leaf pigment concentrations (such as MCARI and NDVI), exhibited significant variations (*P* < 0.001) in all four classification tasks.

**Figure 6:**
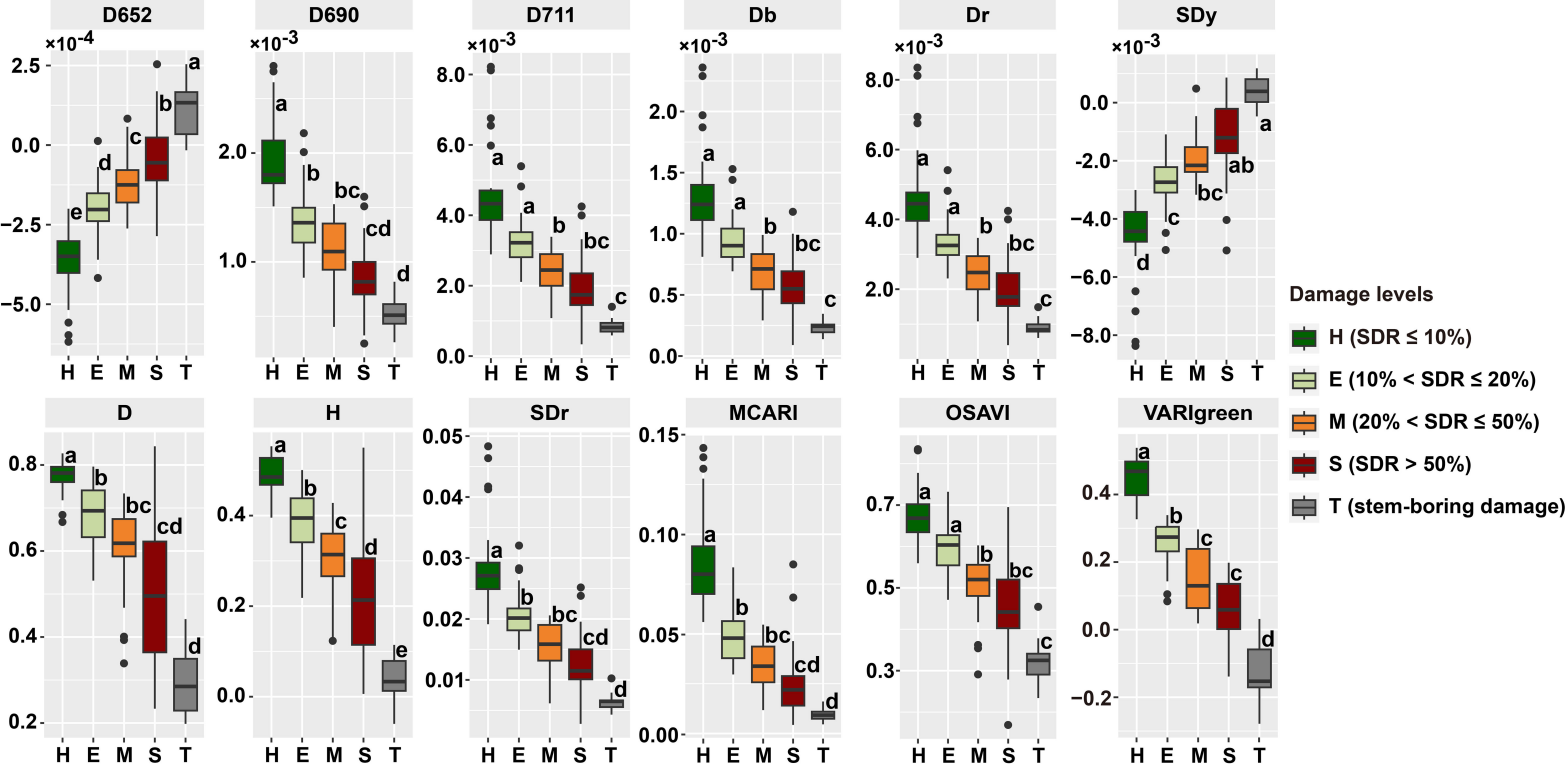
Spectral variables of at different damage levels. The lowercase letters next to the boxplots represent significant difference among different damage levels.

In evaluating the five damage levels, various variables exhibited unique responses to PSB damage (**Figure 6**). Visualization of variables with significant *P* values of difference at the *P* < 0.05 level showed a significant tendency for these variables to decrease or increase as damage levels intensified. Substantial distinctions were observed in the response of nearly all variables to PSB damage fluctuations, particularly at the H and T levels. The variation in response among these variables supports the detection of PSB damage. Variables D652 and H significantly differed among the five damage levels, and variables VARIgreen and SDy significantly differed between T and other levels. The assessment of stem-boring damage (T level) indicated the potential for using pine canopy spectral variables for effective detection.

### 3.3 The importance of variables

**Figure 7:**
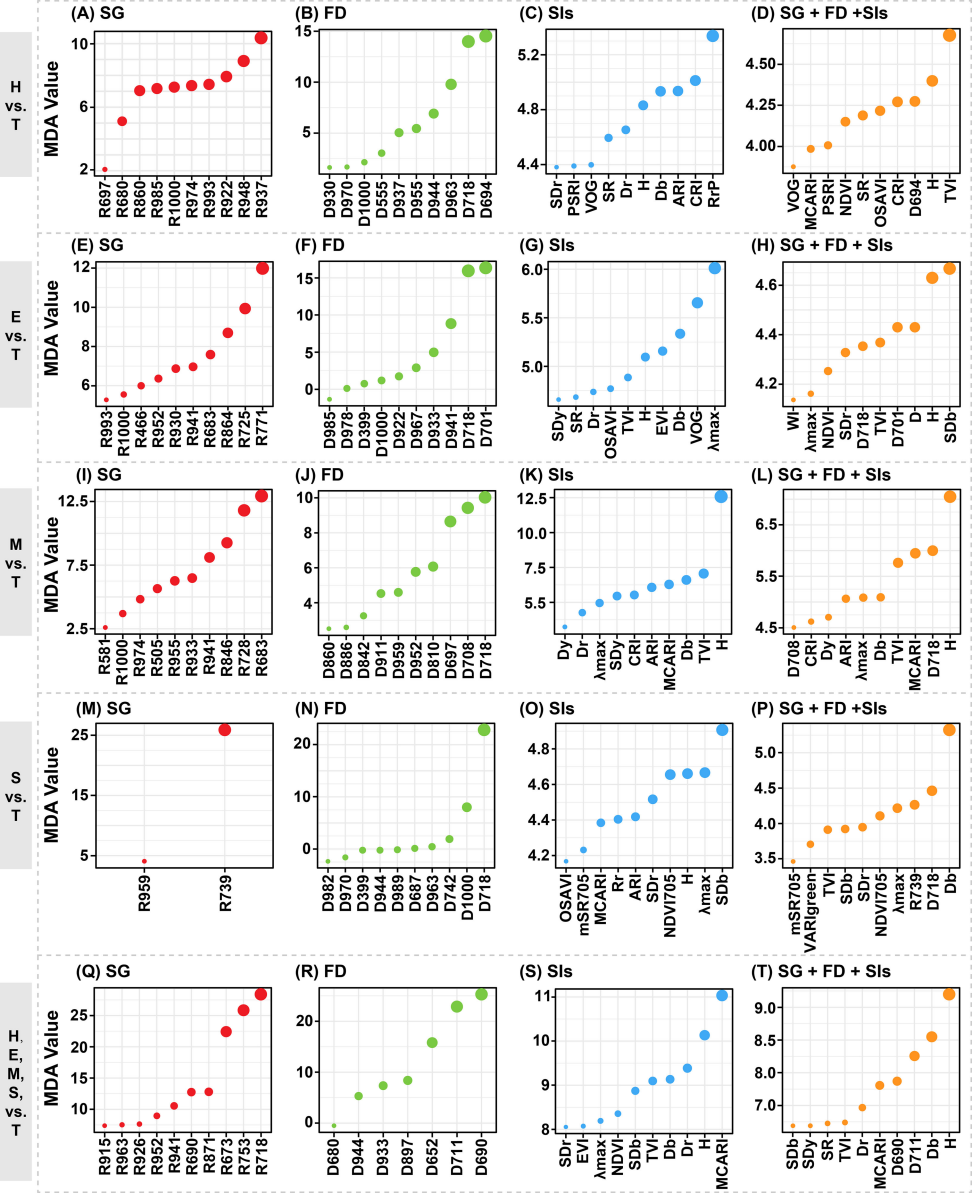
The top ten important spectral variables in detecting the damage levels at the stem-boring stage of pine shoot beetle (*Tomicus* spp.). In cases where the input variables was less than ten, the importance of all variables were showed. Each row represents a classification task, while each column represents an input dataset for a specific category.

We obtained the Mean Decrease Accuracy (MDA) values representing the importance index of each variable through the Random Forest (RF) model. **Figure 6** displayed the MDA values of the top 10 important variables for each model. We compared the MDA values of input variables across each model. However, the MDA values obtained from the RF models using exclusively SG bands, FD bands, or spectral indices (SIs) as input datasets were not directly comparable. Firstly, the importance of input variables was compared across each model. It was observed that in models using exclusively SG bands, the spectral bands around 900 nm were particularly significant. Notably, their importance increased progressively as the disparity between damage levels and the T level widened (**Figures 7A, E, I, M**). This trend was also observed in models using only FD bands (**Figures 7B, F, N, R**). Reflectance near 900 nm is related to water absorption, suggesting a strong correlation between PSB stem-boring damage and canopy water content. Additionally, red edge bands, such as near 700 nm (e.g., D718), were crucial for identifying stem-boring damage, consistently ranking among the top three bands in all comparisons with the T level. In models using only SIs, the variable H was effective in detecting stem-boring damage, ranking in the top five in importance. The spectral indices associated with the blue-edge position, specifically the Db and SDb, demonstrated significant sensitivity in detecting PSB’s stem-boring damage (**Figures 7C, G, K, O**). The combined results of variance analysis and importance assessment of these variables suggest their potential utility as robust indicators for pest infestation evaluation.

Then, the variable importance derived from RF models trained for the classification task involving five damage levels was systematically compared (**Figures 7Q–T**). This analysis enabled the identification of sensitive spectral features, including specific SG bands, FD bands, and SIs, which demonstrated significant potential for distinguishing among the damage levels. In the SG band model, R718 was the most crucial variable, while D690 stood out in the FD band model, and MCARI was prominent in the SIs model. In the combined variable model, the top ten important variables included spectral reflectance and absorbance variables H and D, differential variables such as D690, D711, Db, SDb, SDr, and SDy, and multiple band combinations of SIs like MCARI and TVI associated with changes in chlorophyll content. Most of these variables were in the visible light bands and were strongly linked to changes in canopy color, indicating a significant relationship between PSB damage and canopy chlorosis. Analyzing the combined importance values across all models revealed that canopy spectra’s FDs and SIs were more significant for detecting PSB damage. Specifically, variables like H, D718, and Db showed relative sensitivity to stem-boring damage caused by PSB.

### 3.4 Multivariate-based detection of PSB stem-boring damage

**Figure 8:**
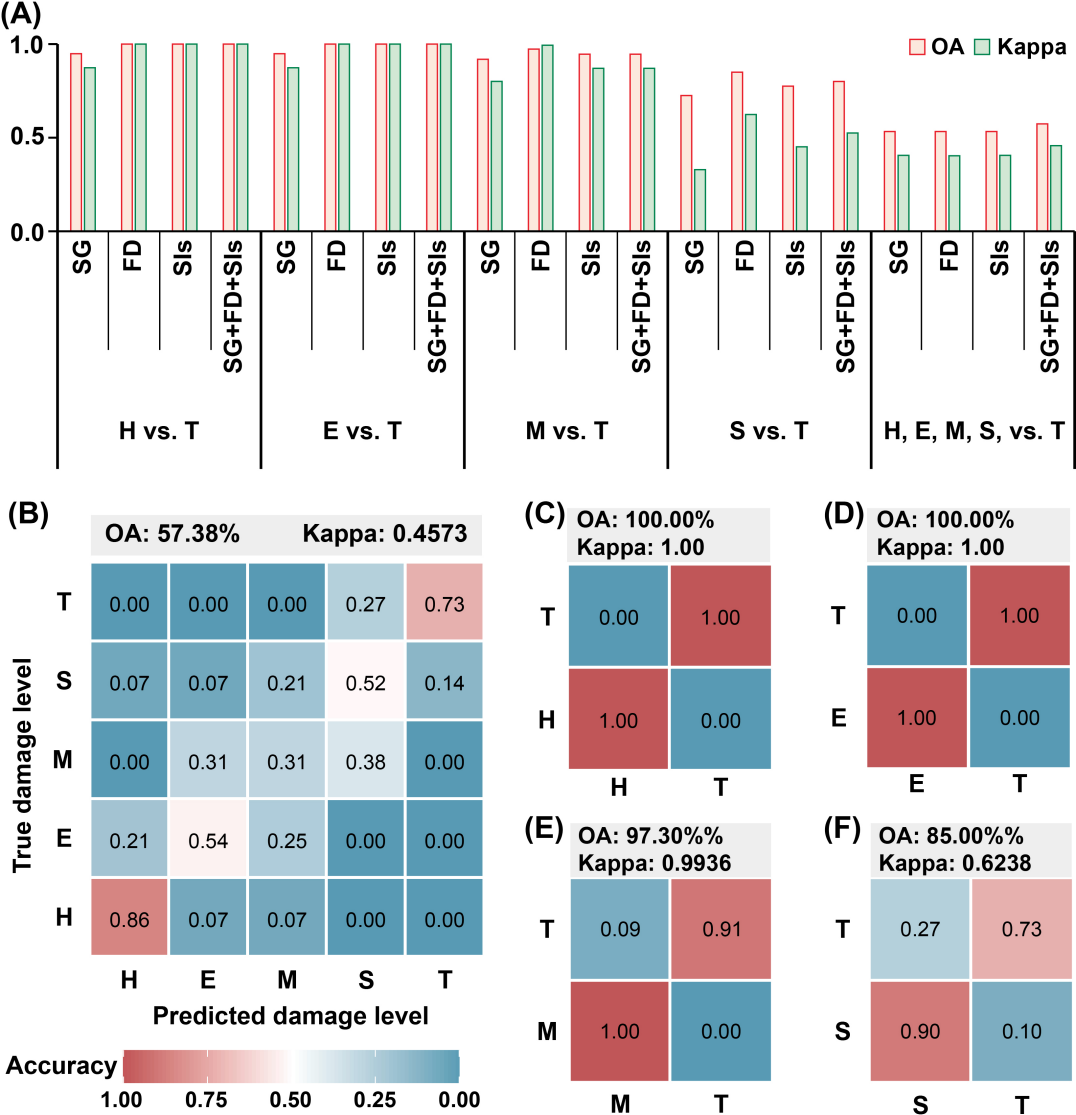
The results of RF model execution. **(A)** The OA and Kappa coefficients of the models for detecting stem-boring damage by pine shoot beetle to Yunan pine based on SG, FD, SIs, and a combination of them. **(B)** Confusion matrix for all damage levels classification. **(C)** Confusion matrix for the classification of H and T levels. **(D)** Confusion matrix for the classification of E and T levels. **(E)** confusion matrix for the classification of M and T levels. **(F)** Confusion matrix for the classification of S and T levels. The numbers in the figure are all rounded.

The OA and Kappa values for each RF model were assessed, as shown in **Figure 8A**. The results indicated that FD bands, SIs, and combined variable models outperformed the SG band model in detecting PSB stem-boring damage, similar to the performance of individual variable models. The combined variables model was particularly effective as the number of damage levels to discriminate increased. Confusion matrices for the best-performing models in each comparison were compiled (**Figures 8B–F**). These included the combined variables model for the H and T levels, the E and T levels, and the five damage levels, as well as the FD bands model for the M and T levels, and the S and T levels. The overall classification accuracy for all damage levels was 57.38%, with a kappa coefficient of 0.4573 (**Figure 8B**). The H level had the highest class accuracy (0.86), followed by the T level (0.73). In contrast, the E, M, and S levels exhibited lower accuracy, with nearly half of the samples in the E and S levels and about 80% of the samples in the M level being misclassified into other levels. For classification between the T level and other damage levels, the H and E levels differed significantly from the T level, achieving an overall accuracy of 100% and kappa coefficients of 1 in both cases (**Figures 8C, D**). The accuracy was slightly lower for the M (**Figure 8E**) and S (**Figure 8F**) levels.

### 3.5 Model compare

**Figure 9:**
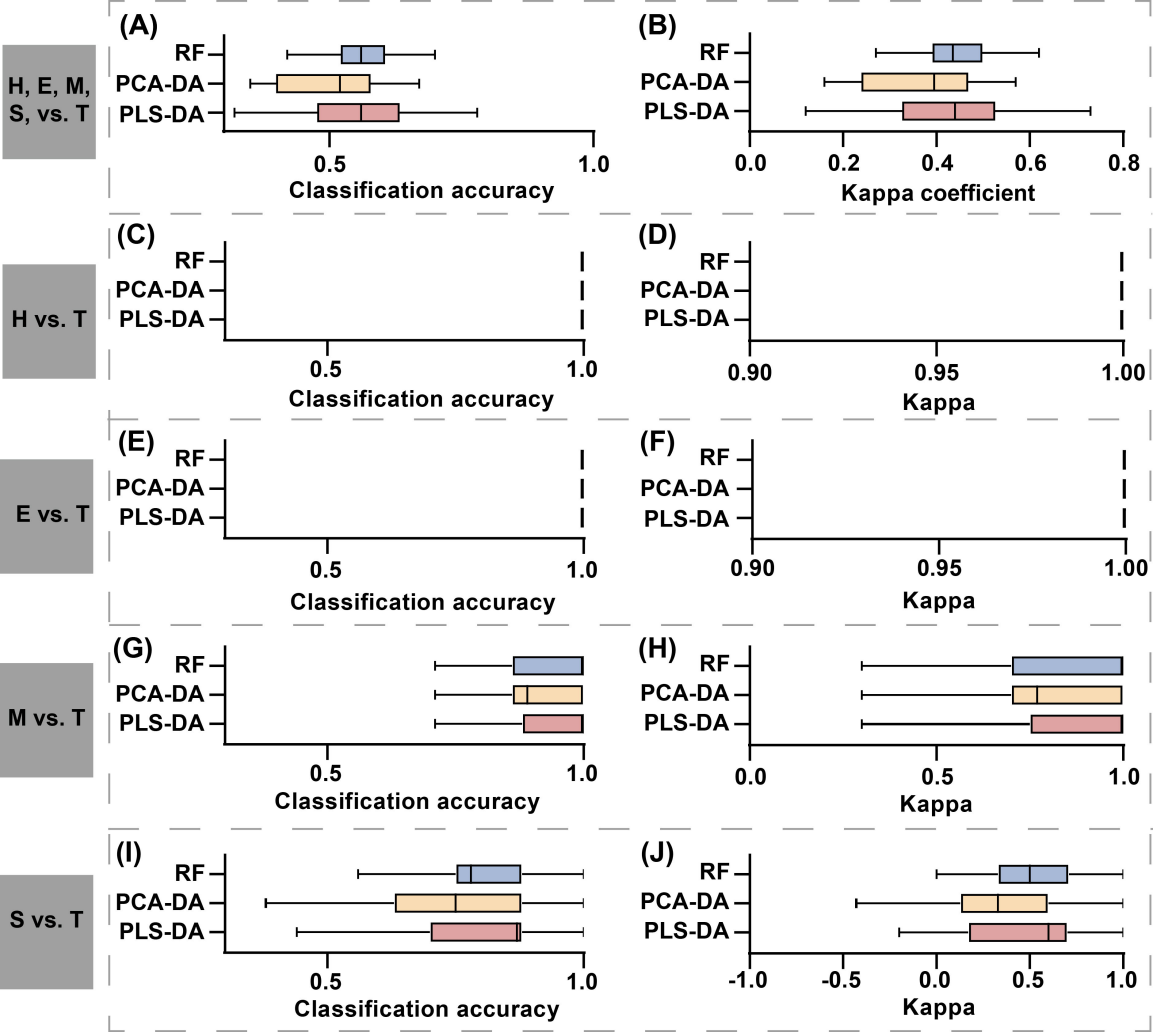
Comparison of classification results among three discriminant models: RF, PLS-DA, and PCA-DA. The first to fifth rows represent the model evaluation results for classification tasks H, E, M, S, vs. T, H vs. T, E vs. T, M vs. T, and S vs. T. The first column shows the model’s OA, and the second column shows the model’s Kappa coefficient. The results showed in the figure represent the aggregated OA and Kappa coefficients obtained from 50 repetitions. In subplots **(C–F)**, both OA and Kappa coefficients consistently achieved a value of 1, which is represented as a vertical line in the figure.

In the model comparison, integrated compositive datasets were consistently used as input data for all models. **Figure 9** presents the classification accuracy and Kappa coefficients obtained from the three classification methods: RF, PLS-DA, and PCA-DA. From the perspective of concentration degree and fluctuation range in classification accuracy and Kappa coefficient distribution, supervised learning methods (RF and PLS-DA) exhibited superior classification performance compared to unsupervised learning approaches (PCA-DA) in detecting of stem-boring damage caused by PSB. Among the supervised learning classification methods, PLS-DA demonstrated superior values in both classification accuracy and Kappa coefficient compared to RF. However, analysis of the distribution patterns revealed that the RF method exhibited greater stability, as evidenced by shorter interquartile ranges and more compact data clustering in boxplot visualizations for both performance metrics (**Figures 9A, B, I, J**). The reduced variability in RF’s classification results suggests its potential advantage in practical applications requiring consistent detection performance.

### 3.6 Differences in the detection of PSB damage levels between shoot-feeding and stem-boring damage stages

**Figure 10:**
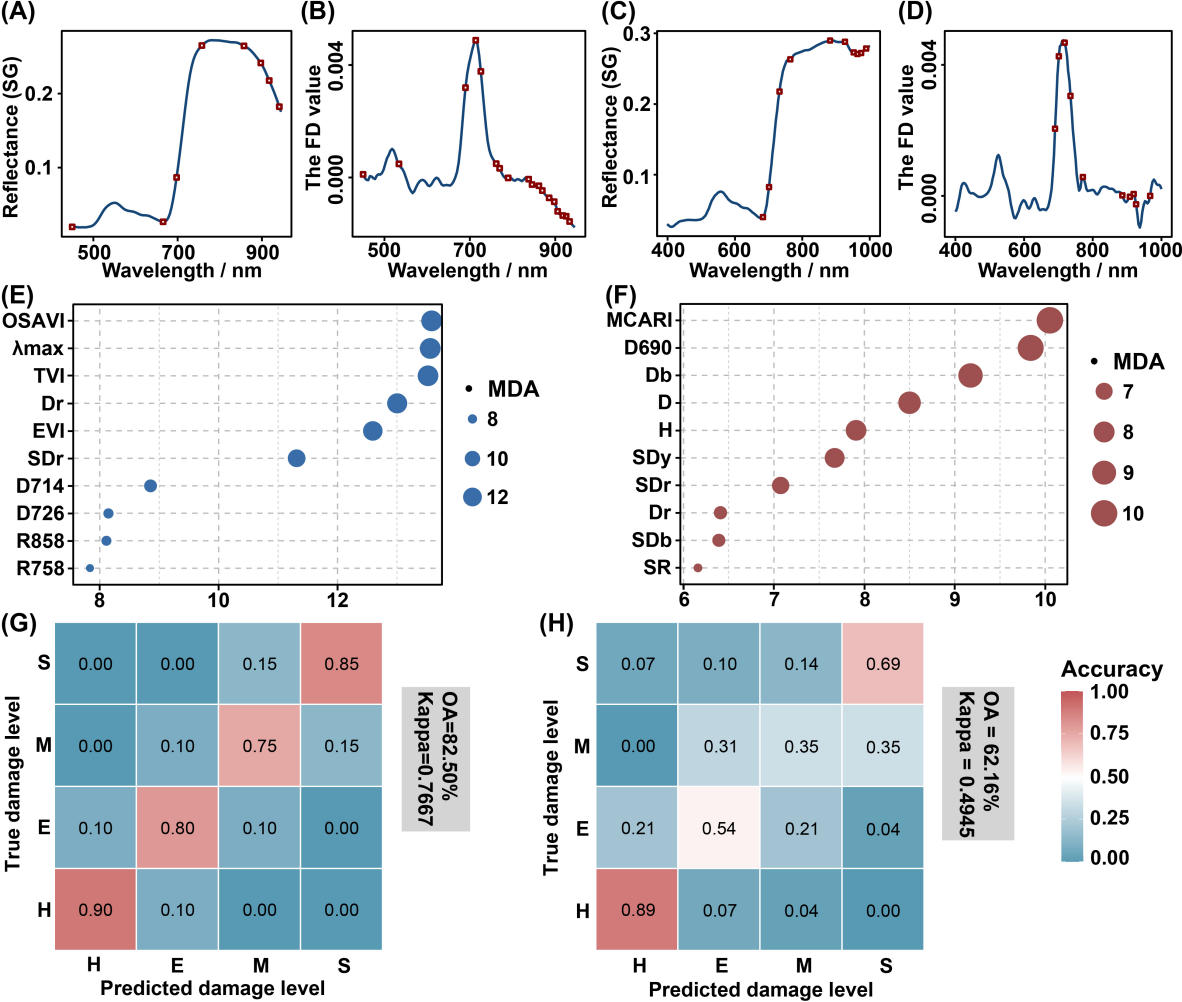
Comparison of the discrimination results between the shoot-feeding and stem-boring damage stages. The results of the feature band screening, variable importance analysis, and RF classification are presented. **(A, B)** are the selected bands of shoot-feeding damage stage, while **(C, D)** are the selected bands of stem-boring damage stage, with the red rectangular boxes indicating the locations of the screened bands. **(E, F)** emphasize the top 10 variables in importance for the shoot-feeding **(E)** and stem-boring **(F)** damage stages. The final two figures illustrate the confusion matrices, showing the classification results for the shoot-feeding **(G)** and stem-boring **(H)** damage stages. The numbers on the confusion matrices were rounded.

The majority of selected feature bands associated with PSB damage at the shoot-feeding and stem-boring stages were concentrated in the red to near-infrared regions, particularly in the red edge region (**Figures 10A–D**). The analysis of variable importance demonstrated that the MDA values of variables at the stem-boring damage stage were generally lower than those at the shoot-feeding damage stage. Bubble plots were used to illustrate the MDA values of the ten most important variables, with OSAVI being identified as the most important at the shoot-feeding damage stage (**Figure 10E**) and MCARI at the stem-boring damage stage (**Figure 10F**). It is noteworthy that SDr and Dr were consistently among the top ten variables for detecting PSB damage in both stages. The classification accuracy was higher for the shoot-feeding damage stage (OA: 82.50%, kappa: 0.7667, **Figure 10G**) compared to the stem-boring damage stage (OA: 62.16%, kappa: 0.4945, **Figure 10H**). The confusion matrices indicated that the H level exhibited the highest accuracy in both stages, followed by the S level. However, higher misclassification rates were observed for the E and M levels.

## 4 Discussion

Given the lethal behavior of PSB predominantly occurring at the stem-boring damage stage, we aimed to assess the potential of canopy hyperspectral data in differentiating the damage levels of PSB at this critical phase. Our results suggest that damaged pine trees displaying stem-boring symptoms can be distinguished based on the distinct spectral features of individual pine canopies. In pairwise comparisons of damage levels H, E, M, and S with the T level, the T level was identified with an accuracy (OA) above 80%. Overall, the accuracy rate for the five damage levels was 57.38%, with the T level showing a 73% accuracy rate. Comparing detection results for PSB damage levels between the shoot-feeding and stem-boring stages revealed significantly higher accuracy at the shoot-feeding damage stage. Variables Dr and SDr emerged as crucial in both stages, underscoring the importance of the red edge feature in the canopy spectrum for detecting PSB’s damage.

### 4.1 The potential of canopy spectra for detecting PSB stem-boring damage

PSB stem-boring damage destroys the phloem and xylem of the stem, cutting off nutrient transport. This lethal behavior typically occurs at the stem-boring damage stage, leading to the rapid death of pine trees (Lieutier et al., 2003; Ye et al., 2004). Our survey indicated that stem-boring damaged pines exhibited more severe damage than healthy and shoot-feeding damaged pines. These pines showed holes on the stem, had shed all needles, and only dry stems remained. Peeling back the bark revealed crisscross galleries (**Figure 1C**).

The changes in pine canopy color, structure, and physiology due to PSB stem-boring damage can be detected in the spectral reflectance characteristics of the canopy. In the visible region, leaf pigmentation primarily controls spectral reflectance (Wong and Gamon, 2015; Falcioni et al., 2020; Zhang et al., 2020). Previous studies have shown that PSB damage decreases chlorophyll content in tree canopies, causing fluctuations in spectral reflectance in the green peak, red valley, and red edge regions (Liu et al., 2020b; Liu et al., 2021). In fact, stem-boring damage significantly affects canopy spectral features associated with pigmentation, leading to decreased reflectance at the green peak and increased reflectance at the red valley. Studies indicate that chlorophyll depletion affects leaf spectral reflectance in the near-infrared region, particularly around 800 nm (Prabhakar et al., 2012; Abdullah et al., 2018; Klouček et al., 2019). Our observations showed a trend of reduced canopy spectra near 800 nm and reduced reflectance around 900 nm, consistent with findings that the 900–970 nm range is sensitive to plant water content (Penuelas et al., 1997).

Various vegetation indices, such as NDVI, SR, EVI, OSAVI, and red edge features, are widely used for detecting plant biotic or abiotic stress (Khodaee et al., 2020; Cañete-Salinas et al., 2024; Zhang et al., 2024). In this study, H, VARIgreen, and red edge features responded rapidly to chlorophyll changes, differentiating between stem-boring damage, shoot-feeding damage, and healthy pines. Additionally, FD features of the canopy spectra, such as D652, Db, and SDb, were sensitive to stem-boring damage. These findings help us better detect PSB stem-boring damage for effective control.

The variable importance analysis showed that FD and SIs features of the canopy spectra were superior to SG features for detecting PSB stem-boring damage, especially in the FD band around 710 nm and SIs related to the blue edge feature (e.g., Db). This finding aligns with the observed variations in canopy spectral features following PSB stem-boring damage. Future research should focus on these specific spectral features to comprehensively detect PSB stem-boring damage.

### 4.2 The detection method for PSB’s stem-boring damage

This study comprehensively described the stem-boring damage caused by PSB and proposed a framework for its detection. Spectral features were determined using the SPA algorithm and SIs construction to reduce computational redundancy. These features were then input into the RF classification model for detecting PSB stem-boring damage. The RF model performed well in pairwise comparisons of the four levels (H, E, M, and S) with the T level for PSB shoot-damage detection. However, accuracy decreased significantly in the joint comparison of all damage levels, primarily due to classification errors in the E, M, and S levels. The model performed best for the H level, followed by the T level. This framework allows for the quick and effective identification of pines with stem damage, aiding in removing damaged trees and preventing the spread of PSB damage in the next shoot-feeding damage stage.

Selecting feature variables from a pool of redundant and complex variables is crucial in spectral analysis as it directly impacts prediction model performance. SPA is commonly used for spectral feature band screening due to its adaptability and capability to address covariance issues (Zhang et al., 2022; Shu et al., 2023). However, as an unsupervised screening method, SPA may lack explanatory power. Future studies should compare other feature selection algorithms, such as Competitive Adaptive Reweighted Sampling, to identify methods that offer superior processing results and faster processing times.

Deep learning (DL) algorithms are increasingly employed in forest pest and disease detection due to their robust computational and learning capabilities, which can enhance detection accuracy (Istiak et al., 2023; Marvasti-Zadeh et al., 2023). However, DL algorithms have specific data requirements, and when data volumes are limited, their performance may not surpass RF and support vector machine (SVM) algorithms in identifying pest-infested trees. Traditional machine learning methods, while capable of achieving high accuracy in detection tasks, are limited in their direct applicability to image data processing. In contrast, advanced deep learning architectures such as Convolutional Neural Networks (CNNs) and You Only Look Once (YOLO) algorithms offer native support for image data analysis. Significant advancements have been made in this domain, as demonstrated by Wang et al. (2024), who developed the YOLO-PWD model based on YOLOv5s model, achieving enhanced accuracy in pine wilt disease (PWD) detection. Furthermore, Feng et al. (2024) proposed the SC-RTDETR, which is primarily structured with Real Time Detection Transformer (RTDETR) and combines modules Soft-threshold and Cascaded-Group-Attention (CGA). Their comparative analysis against the other advanced models including YOLOv8s, YOLOv5s, and RTDETR revealed that SC-RTDETR exhibits superior accuracy and robustness in complex background recognition tasks, particularly in challenging detection scenarios. These findings provide valuable theoretical foundations and methodological references for subsequent research endeavors. Future studies will focus on exploring the potential applications of these advanced algorithms in precision agriculture and forest management, with particular emphasis on developing stronger detection systems for pest and disease damage detection, thereby contributing to sustainable pest and disease control strategies.

### 4.3 Differences between the shoot-feeding and the stem-boring damage stages of PSB

In this study, the accuracy of classifying PSB damage levels at the shoot-feeding stage was notably higher than that at the stem-boring stage, especially for damage levels E, M, and S, where misclassification rates were significantly elevated at the stem-boring stage. The spectral curves for each damage level differed between the two stages. The stem-boring damage stage of PSB in Yunnan coincides with the dry season. Previous research confirmed a consistent decline in the spectra of Yunnan pine forests during this season (Liu et al., 2022). Beyond environmental and equipment factors, the growth characteristics of Yunnan pine significantly contribute to spectral variation between the shoot-feeding and stem-boring damage stages. During the dry season, 2- and 3-year-old needles of Yunnan pine are shed, reducing canopy needle density and affecting the canopy spectrum. Consequently, the spectral characteristics among different damage levels may have become more similar at the stem-boring damage stage.

### 4.4 Error sources

The RF model predicts by aggregating the results of multiple decision tree classifications through voting or averaging (Scornet, 2016). In this study, the RF model showed a higher misclassification rate for the E and M levels of PSB damage. This increased error rate can be attributed to the small sample size, which may cause the prediction results from individual decision trees to be unrepresentative. Additionally, the similarity in spectral features among the E and M damage levels could also contribute to misclassification. It was observed that many pines showed stem-boring damage symptoms were predominantly dead, potentially showing similar canopy spectral characteristics with trees deceased from other causes. To mitigate this interference, the integration of additional discriminative features, such as canopy morphological parameters (Lin et al., 2019), has been proposed. Furthermore, the detection accuracy could enhance through the application of time-series spectral analysis to capture distinctive infestation progression patterns.

## 5 Conclusion

This study conducted a comprehensive spectral analysis distinguishing the stem-boring damage (T level) from the other four levels of shoot-feeding damage caused by PSB, using the hyperspectral data of pine canopy for detecting stem-boring damage. The results showed significant correlations between PSB’s stem-boring damage and specific canopy spectral features, including red edge (such as Dr, SDr, and D711), blue edge (such as Db and SDb), and chlorophyll-related spectral indices (e.g., MCARI). These spectral indicators exhibited strong predictive capabilities for infestation severity, suggesting their potential as reliable biomarkers for early detection of bark beetle damage. Comparative analysis of detection accuracy across damage stages revealed superior performance at the shoot-feeding damage stage compared to the stem-boring damage stage. The findings of this study demonstrate that the optimal period for detecting PSB shoot-feeding occurs at the terminal phase of the shoot-feeding damage stage, rather than at arbitrary time points. These results underscore the critical importance of temporal considerations in pest detection and highlight the limitations of single-time-point assessments for effective PSB damage management. Early detection at shoot-feeding damage stage of PSB facilitates timely intervention to prevent successful stem-boring establishment, while identification of stem-boring damage enables targeted removal of infested wood, thereby mitigating PSB spread during the subsequent shoot-feeding damage stage.

## Data Availability

The raw data supporting the conclusions of this article will be made available by the authors, without undue reservation.
